# A Neuro-Inspired System for Online Learning and Recognition of Parallel Spike Trains, Based on Spike Latency, and Heterosynaptic STDP

**DOI:** 10.3389/fnins.2018.00780

**Published:** 2018-10-31

**Authors:** Gianluca Susi, Luis Antón Toro, Leonides Canuet, Maria Eugenia López, Fernando Maestú, Claudio R. Mirasso, Ernesto Pereda

**Affiliations:** ^1^UCM-UPM Laboratory of Cognitive and Computational Neuroscience, Center for Biomedical Technology, Technical University of Madrid, Madrid, Spain; ^2^Dipartimento di Ingegneria Civile e Ingegneria Informatica, Università di Roma ‘Tor Vergata', Rome, Italy; ^3^Departamento de Psicología Experimental, Procesos Cognitivos y Logopedia, Facultad de Psicología, Universidad Complutense de Madrid, Madrid, Spain; ^4^Departamento de Psicología Clinica, Psicobiología y Metodología, Universidad de La Laguna, La Laguna, Spain; ^5^Instituto de Fisica Interdisciplinar y Sistemas Complejos, CSIC-UIB, Campus Universitat de les Illes Balears, Palma de Mallorca, Spain; ^6^Departamento de Ingeniería Industrial, Escuela Superior de Ingeniería y Tecnología & IUNE, Universidad de La Laguna, La Laguna, Spain

**Keywords:** coincidence detection, spiking neurons, spike latency, delay, heterosynaptic plasticity, STDP, Go/NoGo

## Abstract

Humans perform remarkably well in many cognitive tasks including pattern recognition. However, the neuronal mechanisms underlying this process are not well understood. Nevertheless, artificial neural networks, inspired in brain circuits, have been designed and used to tackle spatio-temporal pattern recognition tasks. In this paper we present a multi-neuronal spike pattern detection structure able to autonomously implement online learning and recognition of parallel spike sequences (i.e., sequences of pulses belonging to different neurons/neural ensembles). The operating principle of this structure is based on two spiking/synaptic neurocomputational characteristics: *spike latency*, which enables neurons to fire spikes with a certain delay and *heterosynaptic plasticity*, which allows the own regulation of synaptic weights. From the perspective of the information representation, the structure allows mapping a spatio-temporal stimulus into a multi-dimensional, temporal, feature space. In this space, the parameter coordinate and the time at which a neuron fires represent one specific feature. In this sense, each feature can be considered to span a single temporal axis. We applied our proposed scheme to experimental data obtained from a motor-inhibitory cognitive task. The results show that out method exhibits similar performance compared with other classification methods, indicating the effectiveness of our approach. In addition, its simplicity and low computational cost suggest a large scale implementation for real time recognition applications in several areas, such as brain computer interface, personal biometrics authentication, or early detection of diseases.

## 1. Introduction

In recent years there has been an increasing interest in applying artificial neural networks to solve pattern recognition tasks. However, it remains challenging to design more realistic spiking neuronal networks (SNNs) which use biologically plausible mechanisms to achieve these tasks (Diehl and Cook, 2015). In sensory systems, the recognition of stimuli is possible by detecting spike patterns during the processing of peripheral inputs. Precise spike timings of neural activity have been observed in many brain regions, including the retina, the lateral geniculate nucleus, and the visual cortex, suggesting that the temporal structure of spike trains serves as an important component of the neuronal representation of the stimuli (Gütig and Sompolinsky, 2006; Zhang et al., 2016). Specific neural mechanisms that recognize time-varying stimuli by processing spiking activity have been an important subject of research (Larson et al., 2010; Masquelier, 2017). Whereas, some investigations are oriented to the study of the spike activity of single neurons, many others consider the timing of spikes across a population of afferent neurons (Gautrais and Thorpe, 1998; Stark et al., 2015).

Plasticity regulates the strength in the connection between neurons. In homosynaptic plasticity the activity in a particular neuron alters the efficacy of the synaptic connection with its target. Instead, in heterosynaptic plasticity changes in the synaptic strength can occur in both stimulated and non-stimulated pathways reaching the same target neuron. Like homosynaptic plasticity, heterosynaptic plasticity has two forms: inhibition and potentiation (Squire, 2013); the latter is not necessarily restricted to a subset of cells, but it can occur to many of the neurons in the population (Han and Heinemann, 2013). A number of distinct subtypes of heterosynaptic plasticity have been found in a variety of brain regions and organisms. They are associated to different neural processes including the development and refinement of neural circuits (Vitureira et al., 2012), extending the lifetime of memory traces during ongoing learning in neuronal networks (Chistiakova and Volgushev, 2009). Among these, heterosynaptic modulation (i.e., when the activity of a modulatory neuron induces a change in the synaptic efficacy between another neuron and the same target cell Phares and Byrne, 2006) allows that one set of inputs exert long-lasting heterosynaptic control over another, enabling the interplay of functionally and spatially distinct pathways (Han and Heinemann, 2013). Among the various types of heterosynaptic plasticity, the heterosynaptic form of Spike-Timing-Dependent Plasticity (STDP) is capturing a lot of interest because recent works have shown that it is a critical factor in the synaptic organization and resulting dendritic computation (Hiratani and Fukai, 2017).

In this paper we introduce a simple but effective network topology specialized in online recognition of temporal patterns. The structure is characterized by lateral excitation, i.e., excitatory connections between neurons that belong to parallel paths, and is based on two features: *heterosynaptic STDP* and *spike latency*. Neurons dynamics is described using the Leaky Integrate-and-Fire with Latency (LIFL) model, which is similar to the Integrate and Fire but supports the spike latency mechanism, extracted from the more realistic Hodgkin-Huxley (HH) model (Salerno et al., 2011). The structure maps spatio-temporal stimuli to specific areas in a temporal, multi-dimensional, feature space. In addition it is able to self-regulate its weights, allowing the learning and recognition of multi-neuronal temporal patterns in parallel spike trains arising from neuronal ensembles. In order to show the potential of the presented structure, we apply it to a cognitive task-recognition problem, considering magnetoencephalografic (MEG) signals of subjects while performing a Go/NoGo task, and compare it with some typical classification methods. The test exhibits good classification performance, indicating the adequateness of our approach.

## 2. Materials and methods

### 2.1. Leaky integrate-and-fire with latency (LIFL) neuron model

The LIFL (Cardarilli et al., 2013; Susi, 2015; Acciarito et al., 2017) is a neuron model similar to the classical Leaky Integrate-and-Fire (LIF), but characterized by the presence of the *spike latency* neurocomputational feature (Izhikevich, 2004; Cristini et al., 2015; Susi et al., 2016). The spike latency is a potential-dependent delay time between the overcoming of the “threshold” and the actual spike generation (Izhikevich, 2004; Salerno et al., 2011). This feature is important because it allows encoding the strength of the input in the spike times (Izhikevich, 2007) extending the neuron computation capabilities over the threshold (e.g., Gollisch and Meister, 2008; Fontaine and Peremans, 2009; Susi, 2015). Neurons with such feature are present in many sensory systems, including the auditory, visual, and somatosensory system (Trotta et al., 2013; Wang et al., 2013). The LIFL neuron model embeds spike latency using a mechanism extracted from the more realistic Hodgkin-Huxley model (Salerno et al., 2011). It is characterized by the internal state *S* (representing the membrane potential),which ranges, for simplicity, from 0 (corresponding to the resting potential of the biological neuron) to ∞.

In its basic implementation, the LIFL model uses a defined threshold (*S*_*th*_), a value slightly greater than 1 that separates two different operating modes: a *passive mode* when *S* < *S*_*th*_, and an *active mode* when *S* > *S*_*th*_. In the passive mode, *S* is affected by a decay, whereas in the active mode it is characterized by a spontaneous growth. For simplicity, we assume that simple Dirac delta functions (representing the action potentials) are exchanged between neurons, in form of pulses or pulse trains.

The LIFL model can be implemented through the event-driven technique (Mattia and Del Giudice, 2000), which provides fast simulations (Ros et al., 2006). When the postsynaptic neuron *N*_*j*_ receives a pulse from the presynaptic neuron *N*_*i*_, its internal state is updated through one of the following equations, depending on whether *N*_*j*_ is in the passive or in the active mode, as:

(1) $$\begin{array}{rcl} S_{{}_{Nj}} & = & S_{p_{Nj}}+A_{{}_{N_{i}}}\cdot w\left(N_{j},N_{i}\right)-T_{l},\text{for }0\leq S_{p_{Nj}}<S_{th} \end{array}$$

(2) $$\begin{array}{rcl} S_{{}_{Nj}} & = & S_{p_{Nj}}+A_{{}_{N_{i}}}\cdot w\left(N_{j},N_{i}\right)+T_{r},\text{for }S_{p_{Nj}}>S_{th} \end{array}$$

*S*_*p*_*Nj*__ represents the postsynaptic neuron's *previous state*, i.e., the internal state immediately before the new pulse arrives. *A*__*N*_*i*___ represents the amplitude of the generated pulse; *w*(*N*_*j*_, *N*_*i*_) represents the *synaptic weight* from neuron *N*_*i*_ to neuron *N*_*j*_. The product *A*__*N*_*i*___·*w*(*N*_*j*_, *N*_*i*_) represents the amplitude of the post-synaptic pulse arriving to *N*_*j*_.

*T*_*l*_, the *leakage term*, accounts for the underthreshold decay of *S* during two consecutive input pulses in the passive mode. We will consider LIFL basic configuration, i.e., characterized by a linear subthreshold decay (as in Mattia and Del Giudice, 2000), where *T*_*l*_ = *L*_*d*_·Δ*t*, being *L*_*d*_ a non negative quantity called *decay parameter* and Δ*t* the temporal distance between two consecutive incoming spikes.

*T*_*r*_, the *rise term*, takes into account the overthreshold growth of *S* during two consecutive input pulses in the active mode. Specifically, once the neuron's internal state crosses the threshold, the neuron is ready to fire. However, firing is not instantaneous, but it occurs after a continuous-time delay. This delay time represents the spike latency, which we call *time-to-fire*, and is indicated with *t*_*f*_ in our model. *t*_*f*_ can be affected by further inputs, making the neuron sensitive to changes in the network spiking activity for a certain period, until the actual spike occurs. *S* and *t*_*f*_ are related through the following relationship, called the *firing equation*:

(3) $$\begin{array}{r} t_{f}=\frac{1}{\left(S-1\right)} \end{array}$$

Such dependence has been obtained through the simulation of a membrane patch stimulated by brief current pulses (0.01 *ms* of duration) and solving the HH equations (Hodgkin and Huxley, 1952) in *NEURON* environment (Hines and Carnevale, 1997), as described in Salerno et al. (2011).

The firing equation is a simple bijective relationship between *S* and *t*_*f*_, observed in most cortical neurons (Izhikevich, 2004); similar behaviors have been found by other authors, such as Wang et al. (2013) and Trotta et al. (2013), using DC inputs.

The firing threshold is written as:

(4) $$\begin{array}{r} S_{th}=1+d \end{array}$$

where *d* is a positive value called *threshold constant*, which fixes a bound for the maximum value of *t*_*f*_. According to Equation 4, when *S* = *S*_*th*_, *t*_*f*_ is maximum, and equals to:

(5) $$\begin{array}{r} t_{f,max}=1/d \end{array}$$

*t*_*f, max*_ represents the upper bound of the time-to-fire and is a measure of the finite maximum spike latency of the biological counterpart (FitzHugh, 1955).

Under proper considerations (see section 1 of Supplementary material), it is possible to obtain *T*_*r*_ (*rise term*), as follows:

(6) $$\begin{array}{r} T_{r}=\frac{{\left(S_{p}-1\right)}^{2}\Delta t}{1-\left(S_{p}-1\right)\Delta t}. \end{array}$$

**Figure 1:**
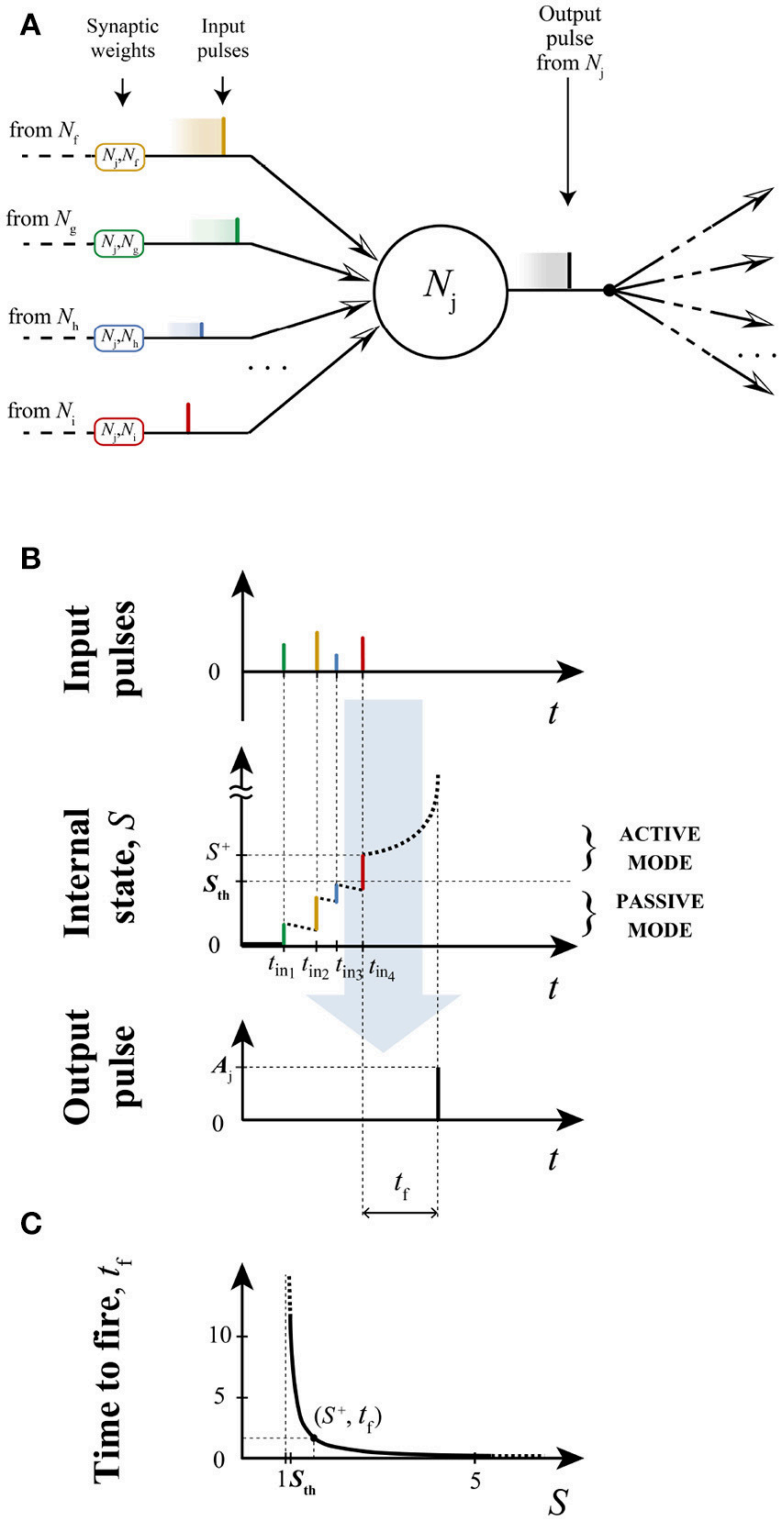
Neural summation and spike generation in a LIFL neuron. **(A)** Input/output process scheme; **(B)** temporal diagram of LIFL operation (basic configuration), assuming the neuron starts from its resting potential. For simplicity contributions are supposed to be all excitatory so that each incoming input causes an instantaneous increase of the internal state. In the passive mode the neuron is affected by a decay; when *S* exceeds the threshold (*S* = *S*^+^) the neuron is ready to spike; due to the latency effect, the firing is not instantaneous but it occurs after a time *t*_*f*_. Once emitted, the pulse of amplitude *A*_*Nj*_ is routed to all the subsequent connections, and then multiplied by the related weight. In **(C)** is shown the firing equation, i.e., the latency curve for the determination of *t*_*f*_ from *S*^+^(see Salerno et al., 2011). In this case *d* is set to 0.04.

*S*_*p*_ represents the neuron's previous state, and Δ*t* is the temporal distance between two consecutive input pulses. Equation 6 allows us to determine the internal state of the postsynaptic neuron at the time that it receives further inputs during the *t*_*f*_ time window. In Figure 1, the operation of LIFL is illustrated. Neurons are supposed to interact instantaneously, through the *synaptic weight* *w*(*N*_*j*_, *N*_*i*_). Such link element can introduce amplification/attenuation of the traveling pulse.

The operation of the LIFL model is illustrated in Figure 1. Note that in this, and following figures, the synaptic weight is displayed with rounded rectangles, and identified by its post- and pre- synaptic neurons respectively. For a given neuron *N*_*j*_ operating in the active mode, the arrival of new input pulses updates the time-to fire *t*_*f*_. If no other pulse arrives during this interval, the output spike is generated and *S* is reset. The presented basic configuration of the LIFL model defines an intrinsically *class 1 excitable, integrator* neuron, supporting *tonic spiking* and *spike latency*. We also included in the neuron model the *absolute refractory period*, for which after the spike generation, the neuron's internal state remains at zero for a period *t*_*arp*_, arbitrarily set. During this period the neuron becomes insensitive to further incoming spikes.

### 2.2. Spike-timing-dependent plasticity (STDP)

STDP is a well-known type of plasticity consisting of an unsupervised spike-based process that can modify the weights on the basis of network activity. It underlies learning and information storage in the brain, and refines neuronal circuits during brain development (Sjöström and Gerstner, 2010). The STDP mechanism influences the synaptic weights on the basis of the difference between the time at which the pulse arrives at the presynaptic terminal and the time a pulse is generated in the postsynaptic neuron.

The original STDP behavior (Bi and Poo, 1998) can be modeled by two exponential functions (Abbott and Nelson, 2000).

(7) $$\begin{array}{l} \left\{ \begin{array}{l} \Delta W=A_{+}{\text{e}}^{-\frac{\Delta T}{\tau_{+}}},\text{for }\Delta T>0\;\\ \Delta W=0,\text{for }\Delta T=0\;\\ \Delta W=A_{-}{\text{e}}^{\frac{\Delta T}{\tau_{-}}},\text{for }\Delta T<0\;\\ \; \end{array}\right. \end{array}$$

where Δ*T* is the difference between the time at which the postsynaptic neuron fires (i.e., *t*_*post*_) and the time at which the pulse arrives at the presynaptic terminal (i.e., *t*_*pre*_):

(8) $$\begin{array}{r} \Delta T=t_{post}-t_{pre} \end{array}$$

**Figure 2:**
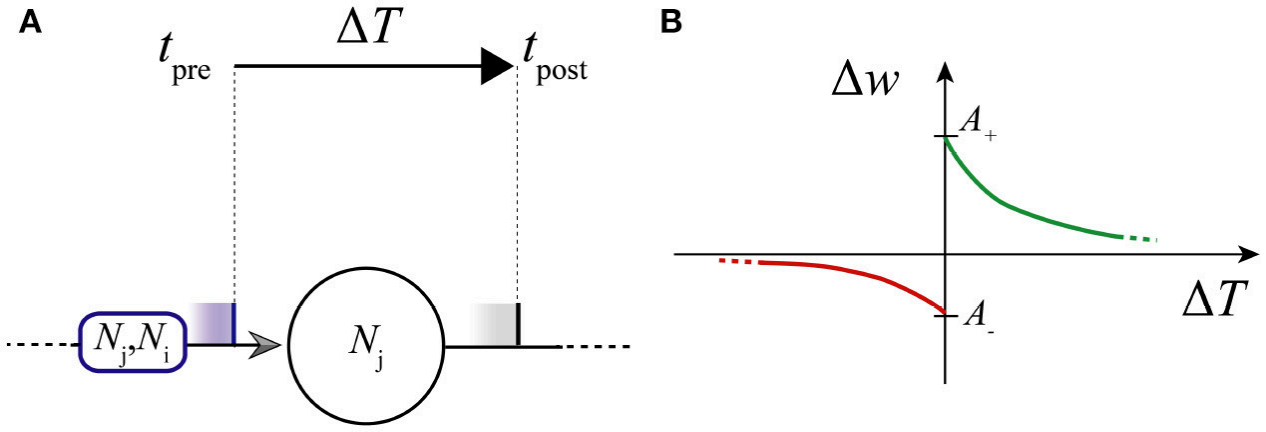
Scheme of STDP. **(A)** Δ*T*; **(B)** Learning window: LTD and LTP curves (in red and green, respectively).

τ_+_ and τ_−_ are positive time constants for *long-term potentiation* (LTP, Equation 7a) and *long-term depression* (LTD, Equation 7c), respectively; *A*_+_ and *A*_−_ (positive and negative values, respectively) are the maximum amplitudes of potentiation and depression which are chosen as absolute changes, as in other works (e.g., Acciarito et al., 2017). Then, a weight is increased or decreased depending on the pulse order (*pre-*before *post-*, or *post-* before *pre-*, respectively; see Figure 2).

As explained in section 2.1, in the LIFL model the delay in generating an output spike (spike latency) depends on the value of the internal state reached by the neuron. Since the STDP can modulate the amplitude of the neuron inputs, the combination of STDP and LIFL makes possible to implement a form of *delay learning* (Taherkhani et al., 2015), i.e., the modulation of the delay to achieve learning.

In this work we will focus on heterosynaptic STDP plasticity, by which the Δ*T* referred to a given synaptic afferent determines the modification of other synaptic afferents to the same neuron (Phares and Byrne, 2006), enabling the interplay of distinct pathways of the same structure.

### 2.3. Multi-neuronal spike sequence detector

A broad range of literature is aimed at understanding how animals have the capability to learn external stimuli and to refine its internal representation. Many of these studies propose architectures based on delays and coincidence detection mechanisms (König et al., 1996; Hedwig and Sarmiento-Ponce, 2017).

**Figure 3:**
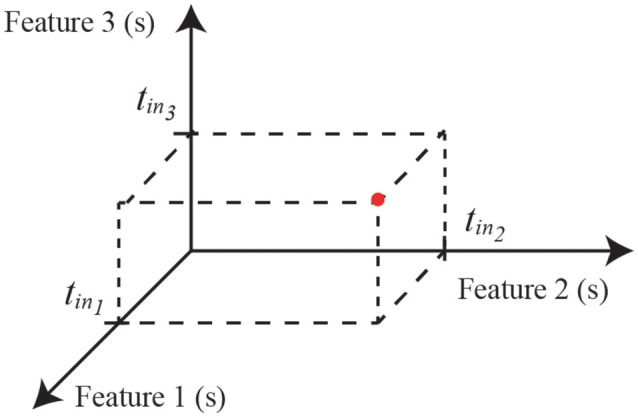
An object characterized by three features can be identified in a three-dimensional feature space by the arrival times of three input pulses. In this way, given a multi-neuronal spike sequence as input, the MNSD will associate it to the represented class whenever the input spikes fall in proper temporal ranges.

In a classic pattern recognition problem an object can be described by a n-dimensional vector (or matrix) where each component represents an object's feature. Analogously, in the neural computation context, an object can be identified by an n-uple of pulses, where feature attributes are encoded in the times at which the pulses occur (Susi, 2015). This allows us to map the classes in a n-dimensional topological space of the internal object representation (see Figure 3).

We present here a multi-neuronal spike pattern detector that includes a bio-plausible self-tuning mechanism, which is able to learn and recognize multi-neuronal spike sequences through repeated stimulation, without supervision. We term this neuromorphic structure as *Multi-Neuronal spike-Sequence Detector* (MNSD). Through a MNSD we can mediate the mapping from spatio-temporal stimuli to such temporal feature space, identifying a class with a specific area, which we call *class hypervolume*. In this section we show the operation principles on which such structure is based.

#### 2.3.1. Structure description

**Figure 4:**
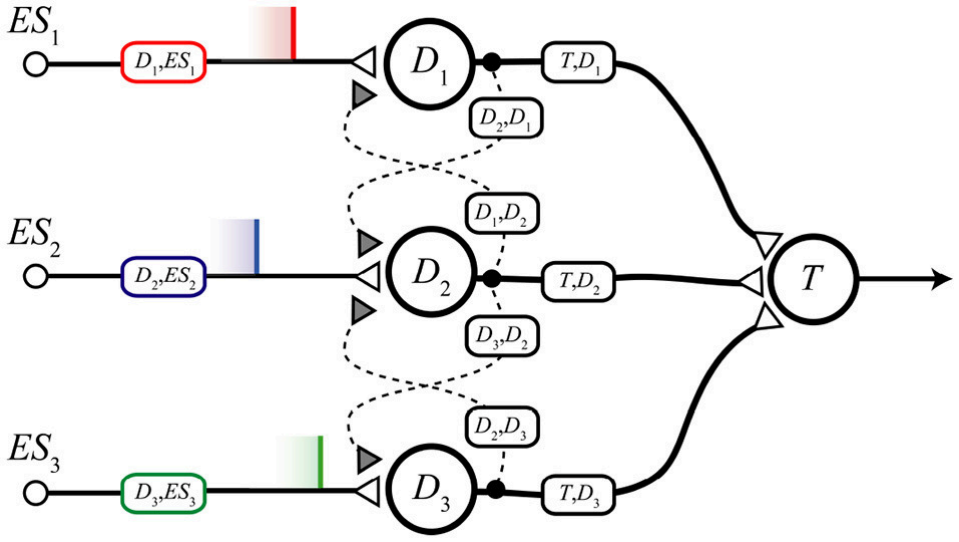
Scheme of the presented structure. The three delay branches, characterized by the three delay neurons *D*_1_, *D*_2_, *D*_3_, converge to the target neuron *T*. Heterosynaptic STDP interactions are permitted by lateral connections, represented as dotted curves with related synapses.

The MNSD architecture, represented in Figure 4, is composed of:

- a layer of neurons *D*_1_, …, *D*_*n*_ (termed *delay neurons*) which receive the external spikes *ES*_*n*_ and are subject to heterosynaptic STDP interactions between them. For simplicity we only consider nearest-neighbor interactions between delay neurons, i.e., each branch can interact with its neighbors branches only (in order to mimic a layer of adjacent neurons).
- one target neuron *T*, which performs the summation of previous contributions and acts as readout neuron, signaling the recognition of the sequence.

To facilitate the analysis and to map the spatio-temporal stimuli in three dimensions we will consider a structure with only three branches; nevertheless, we can generalize to structures of as many branches as features of the object we want to classify. We also consider that the interactions between the neurons are instantaneous; then the only possible delays in the network are those produced by the spike latency.

In order to perform the recognition, the structure's weights *w*(*D*_*n*_, *ES*_*n*_), (i.e., the efficacies of the synapses projecting from *ES*_*n*_ to *D*_*n*_) are adaptively adjusted on the basis of the specific mutineuronal spike sequence given at the input. In this way the target neuron (*T*) will become active only at the presentation of such sequence (or similar ones, as we will see in section 2.3.3).

The necessary condition for *T* to spike is that *S* > *S*_*th*_; this is made possible by the synchronization of the (excitatory) contributions coming from the delay branches. Synchronizability at the target neuron in response to the specific sequence is progressively obtained through the repeated presentation of the sequence to the structure, due to the interplay between the spike latency and the heterosynaptic STDP. Through the amplitude-time transformation operated by the spike latency feature it is possibile to obtain synchronization on the target neuron acting on the amplitude of the pulses at the input of the delay neurons. The spike latency feature is then fundamental for the correct operation of the structure (a simple LIF would not be able to support this mechanism). The interaction between adjacent branches (lateral excitation) combined with the hetherosynaptic STDP make it possible *w*(*D*_*n*_, *ES*_*n*_) to change with respect to the difference between their spike times. This modifies the amplitudes of the contributions in the input of the different branches, enabling a feedback mechanism to mutually compensate the differences between the output spike times of adjacent branches and to produce a synchronous arrival to the target.

With the aim of better explaining the operation of the MNSD structure, we initially perform an analysis of the structure without plasticity (i.e., *static analysis*). Later, we will include a (hetero-)synaptic term to show how one branch can adapt dynamically to reach a downstream spike synchrony with its neighbor (*dynamical analysis*). In order to design structures that are capable to face real problems by operating with this principle, we will derive the set of relations in sections 2.3.2 and 2.3.3, and then we tune a MNSD for a specific application (section 3).

#### 2.3.2. Static analysis

**Figure 5:**
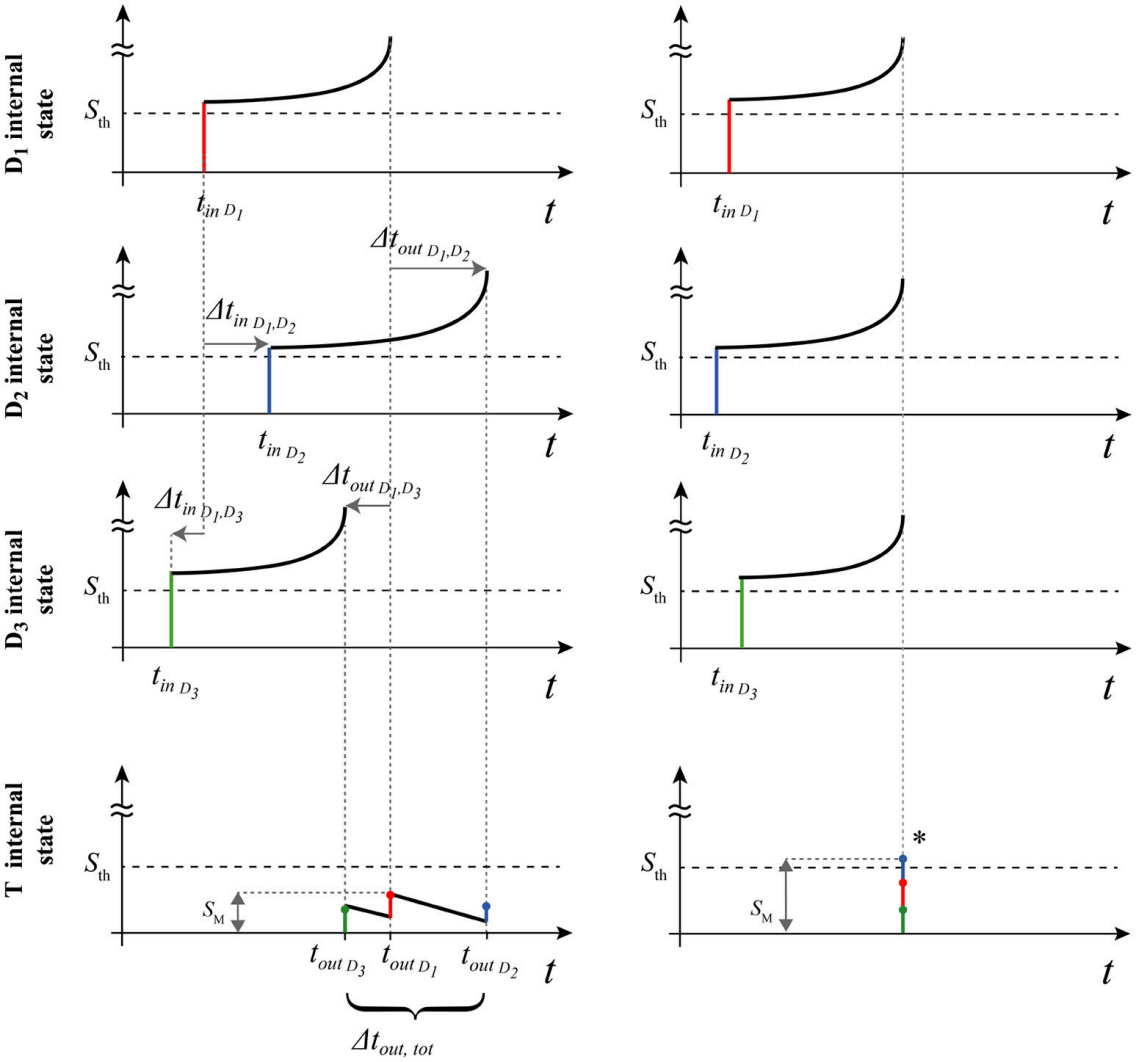
Diagram of the operating principle of the structure (static analysis). On the left, desynchronized input pulses are unable to activate the target. Note that depending on the arrival orders of *t*_*in*_ (and *t*_*out*_), some Δ_*in*_ (and Δ_*out*_) can assume negative values (arrow directions are significant). At right, *simultaneity condition* allows the target activation. Finally, maximum state *S*_*M*_ is represented for both the cases.

In this section we obtain the conditions that allows *T* to generate a spike, without considering the plastic term (i.e., not considering the dotted connections of Figure 4). The operation of the structure in the static mode is shown by means of the temporal diagrams in Figure 5.

The excitatory neurons *D*_*n*_ , present in the afferent branches, allow to create a transmission delay through the spike latency mechanism. The operation is based on the fact that the pulses arriving from the different branches can evoke a spike in *T* only if they arrive sufficiently close in time.

In the following we indicate with *t*_*i*_*n*__*D*_*n*___ the arrival instant of the external spike *ES*_*n*_ and with *t*_*ou*_*t*__*D*_*n*___ the time at which the output pulse of *D*_*n*_ is generated; Δ*t*_*i*_*n*__*D*_*m*_, *D*_*n*___ represents the time difference between the pulses afferent to the delay neurons (i.e., *t*_*i*_*n*__*D*_*n*___−*t*_*i*_*n*__*D*_*m*___), and Δ*t*_*ou*_*t*__*D*_*m*_, *D*_*n*___ the time difference between the pulses afferent to the target neuron (i.e., *t*_*ou*_*t*__*D*_*n*___−*t*_*ou*_*t*__*D*_*m*___). Let us consider the amplitude of the pulses. At the input, and to guarantee the activation of *D*_*n*_, the following relation has to be satisfied:

(9) $$\begin{array}{r} A\left(ES_{n}\right)\cdot w\left(D_{n},ES_{n}\right)\geq 1+d \end{array}$$

where *A*(*ES*_*n*_) is the amplitude of the external spike, *w*(*D*_*n*_, *ES*_*n*_) the synaptic weight afferent to *D*_*n*_, and their product represents the amplitude of the input pulse arriving to *D*_*n*_. For simplicity we consider that:

- neurons are identical, i.e., initialized with the same set of parameters presented in section 2.1;
- synaptic weights afferent to the target are the same for the three afferent connections: *w*(*T, D*_1_) = *w*(*T, D*_2_) = *w*(*T, D*_3_) = *w*(*T, D*_*n*_)
- External spikes *ES* , as well as output pulses, are assumed to have the same amplitude [*A*(*ES*_*n*_) = 1]

Then:

(10) $$\begin{array}{r} w\left(D_{n}\right)\geq 1+d \end{array}$$

Assuming that the pulses arrive simultaneously at the target (simultaneity condition), we have that the following relation has to be satisfied to guarantee the output spike of neuron *T*:

(11) $$\begin{array}{r} w\left(T,D_{n}\right)\geq \frac{1+d}{3} \end{array}$$

In order to have the target activated with the contribution of all the three branches (avoiding that the target neuron generates a spike also for partial sequences that do not exhibit the whole set of features of our object), we have the following constraint:

(12) $$\begin{array}{r} w\left(T,D_{n}\right)<\frac{1+d}{2} \end{array}$$

Now we introduce the delay times due to the spike latency. Considering Figure 5, we can write the system of equations that relates the arrival times of the three contributions to *T* as:

(13) $$\begin{array}{l} \left\{ \begin{array}{l} t_{f}\left(D_{1}\right)+\Delta t_{out_{D_{1},D_{2}}}=\Delta t_{in_{D_{1},D_{2}}}+t_{f}\left(D_{2}\right)\;\\ t_{f}\left(D_{1}\right)+\Delta t_{out_{D_{1},D_{3}}}=\Delta t_{in_{D_{1},D_{3}}}+t_{f}\left(D_{3}\right)\; \end{array}\right. \end{array}$$

In order to achieve simultaneous arrival of the pulses to the target, we should have:

(14) $$\begin{array}{r} \Delta t_{out_{D_{m},D_{n}}}=0. \end{array}$$

Then:

(15) $$\begin{array}{l} \left\{ \begin{array}{l} t_{f}\left(D_{1}\right)=\Delta t_{in_{D_{1},D_{2}}}+t_{f}\left(D_{2}\right)\;\\ t_{f}\left(D_{2}\right)=\Delta t_{in_{D_{1},D_{3}}}+t_{f}\left(D_{3}\right)\; \end{array}\right. \end{array}$$

This means that, for a simultaneous arrival of pulses at the target, with the above-mentioned restrictions, we should have:

(16) $$\begin{array}{l} \left\{ \begin{array}{l} \Delta t_{in_{D_{1},D_{2}}}=\frac{1}{w\left(D_{1},ES_{1}\right)-1}-\frac{1}{w\left(D_{2},ES_{2}\right)-1}\;\\ \Delta t_{in_{D_{1},D_{3}}}=\frac{1}{w\left(D_{1},ES_{1}\right)-1}-\frac{1}{w\left(D_{3},ES_{3}\right)-1}\; \end{array}\right. \end{array}$$

Now we remove the simultaneity condition at the target, searching for the values of Δ*t*_*in*_ and *w*(*T, D*_*n*_) for which the spike at the target neuron is still allowed. Under proper considerations (see section 2 of Supplementary material), we arrive to the following relations:

- If Δ*t*_*i*_*n*__*D*_1_, *D*_2___ and Δ*t*_*i*_*n*__*D*_1_, *D*_3___ have concordant sign, then:
  (17) $$\begin{array}{l} max(|\Delta t_{in_{D_{1},D_{2}}}-\frac{1}{w(D_{1},ES_{1})-1}+\frac{1}{w(D_{2},ES_{2})-1}|,|\Delta t_{in_{D_{1},D_{3}}}\\ \;-\frac{1}{w(D_{1},ES_{1})-1}+\frac{1}{w(D_{3},ES_{3})-1}|)<\frac{2-d}{L_{d}} \end{array}$$
- On the contrary, if Δ*t*_*i*_*n*__*D*_1_, *D*_2___ and Δ*t*_*i*_*n*__*D*_1_, *D*_3___ have discordant sign, then:
  (18) $$\begin{array}{l} \;|(\Delta t_{in_{D_{1},D_{2}}}-\frac{1}{w(D_{1},ES_{1})-1}+\frac{1}{w(D_{2},ES_{2})-1})\\ -(\Delta t_{in_{D_{1},D_{3}}}-\frac{1}{w(D_{1},ES_{1})-1}+\frac{1}{w(D_{3},ES_{3})-1})|<\frac{2-d}{L_{d}} \end{array}$$

If we aim at recognizing parallel spike trains of greater cardinality, it is necessary to increase the number of delay branches, keeping the condition that the contributions have to arrive simultaneously to the target neuron.

#### 2.3.3. Dynamical analysis

As already mentioned, the operational key of the structure resides in the interplay of spike latency and plasticity: the delay in neuronal pathways is due to the spike latency, which in turn depends on *w*(*D*_*n*_, *ES*_*n*_). In addition *w*(*D*_*n*_, *ES*_*n*_) is modulated by the neighbor branch(es) through heterosynaptic plasticity. Therefore, the branch delay is modulated by plasticity. In the presence of plasticity and under repetitive stimulation, the structure can progressively self-regulate its weights until the multi-neuronal spike train synchronizes in the target neuron (operation mode described in the previous section).

**Figure 6:**
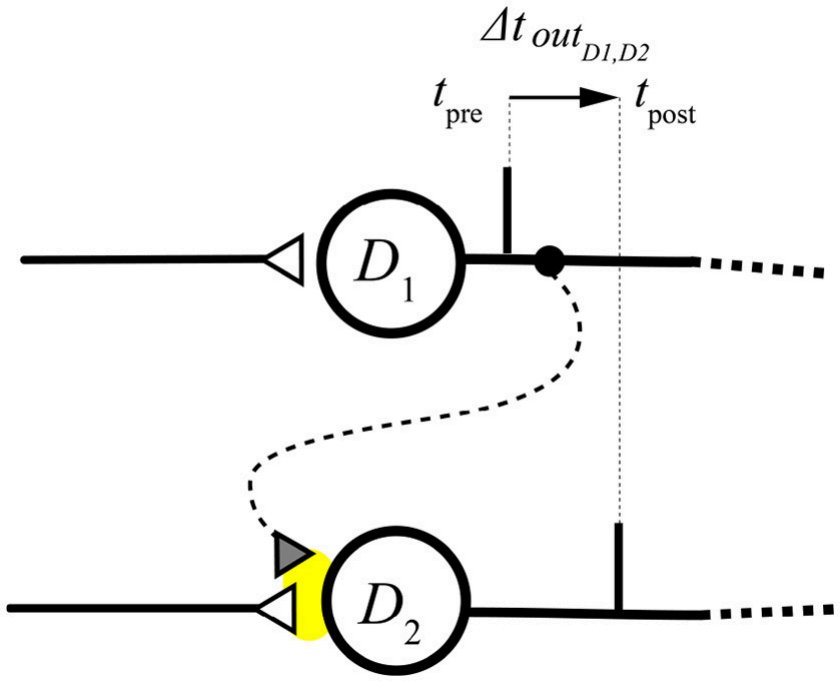
Lateral excitatory heterosynaptic junction. The area of synapse modification is highlighted in yellow.

For simplicity, and without loss of generality, we consider here the effect of a single heterosynaptic connection (the influence of a single branch on an adjacent one). In the whole structure, however, each branch acts on its neighbors through heterosynaptic lateral junctions. This leads to a modification of the timing of the branch's pulse in order to converge to the neuron *T* temporally closer with respect to their neighbor(s). Such mechanism is shown in Figure 6 where heterosynapsis is indicated with a dotted curve and a gray triangle. In this way the weight *w*(*D*_2_, *ES*_2_) is modulated by the time difference between the output pulse of *D*_2_ and the contribution from *D*_1_ (i.e., the output pulse of *D*_1_). In the case of generic heterosynaptic plasticity, the weight potentiation/depression will involve all the afferences of *D*_2_, but if we assume that the lateral contribution is weak, its impact on the inner state of *D*2 will be negligible, then the modifications related to the input *w*(*D*_2_, *ES*_2_) will be the relevant ones for the operation of the structure, and lateral contributions will have only a modulatory function (heterosynaptic modulation).

Considering that connections are instantaneous (as specified in section 2.3), we note that the Δ*T* cited in section 2.2 in this configuration corresponds to Δ*t*_*out*_*D*__1_, *D*_2__ (see Figure 6). We can then write:

(19) $$\{\begin{array}{l} \Delta w(D_{2},ES_{2})=A_{+}{\text{e}}^{-\frac{\Delta t\;out_{D_{1},D_{2}}}{\tau_{+}}},\text{ for }\Delta t\;out_{D_{1},D_{2}}>0\\ \Delta w(D_{2},ES_{2})=0,\text{ for }\Delta t\;out_{D_{1},D_{2}}=0\\ \Delta w(D_{2},ES_{2})=A_{-}{\text{e}}^{\frac{\Delta t\;out_{D_{1},D_{2}}}{\tau_{-}}},\text{ for }\Delta t\;out_{D_{1},D_{2}}<0 \end{array}$$

The difference Δ*t* *out*_*D*_1_, *D*_2__ elicits an increase of the weight *w*(*D*_2_, *ES*_2_) when the arrival pulses order is *D*_2_, *D*_1_ (a decrease otherwise), causing a decrease (increase) of the latency at the arrival of the next *ES*_2_.

Considering now the overall structure, we have to take it into account for the generic *D*_*n*_ the influence of both the neighboring branches (i.e., the influence of *D*_*n*−1_, and that of *D*_*n*+1_, on *D*_*n*_). In this way, for the update of the weight *w*(*D*_*n*_, *ES*_*n*_) the following two sets of equations are used:

- influence of *D*_*n*−1_ on *D*_*n*_
  (20) $$\left\{ \begin{array}{l} \Delta w(D_{n},ES_{n})=A_{+}e^{-\frac{\Delta t\;out_{D_{n-1},D_{n}}}{\tau_{+}}},\;for\;\Delta t\;out_{D_{n-1},D_{n}}>0\\ \Delta w(D_{n},ES_{n})=0,\;for\;\Delta t\;out_{D_{n}-1,D_{n}}=0\\ \Delta w(D_{n},ES_{n})=A_{-}e^{\frac{\Delta t\;out_{D_{n-1},D_{n}}}{\tau_{-}}},\;for\;\Delta t\;out_{D_{n-1},D_{n}}<0 \end{array}\right.$$
- influence of *D*_*n*+1_ on *D*_*n*_
  (21) $$\left\{ \begin{array}{l} \Delta w(D_{n},ES_{n})=A_{+}e^{-\frac{\Delta t\;out_{D_{n+1},D_{n}}}{\tau_{+}}},\;for\;\Delta t\;out_{D_{n+1},D_{n}}>0\\ \Delta w(D_{n},ES_{n})=0,\;for\;\Delta t\;out_{D_{n}+1,D_{n}}=0\\ \Delta w(D_{n},ES_{n})=A_{-}e^{\frac{\Delta t\;out_{D_{n+1},D_{n}}}{\tau_{-}}},\;for\;\Delta t\;out_{D_{n+1},D_{n}}<0 \end{array}\right.$$

These equations apply to all delay neurons except for the first and the last branches, which have only one neighbors.

Synaptic changes must be induced by spikes belonging to the same sequence. Consequently, it is important to prevent interference between subsequent multi-neuronal sequences. This is done by carefully adjusting the STDP time constants.

**Figure 7:**
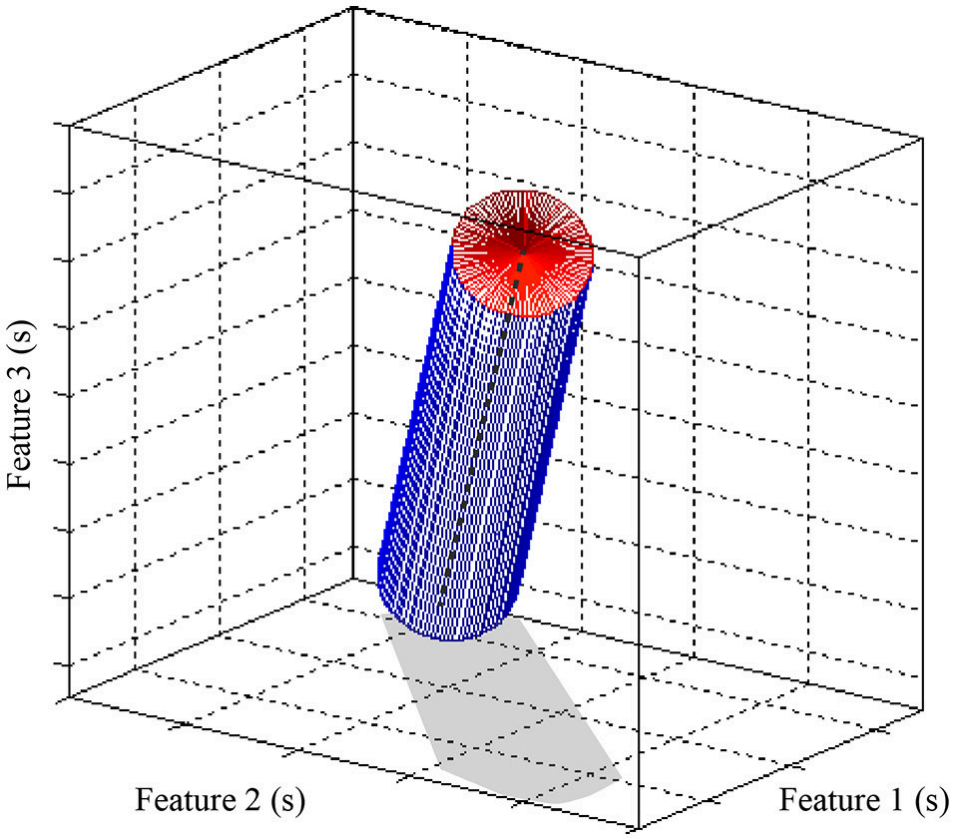
Representation of class hypervolume identified by a three dimensional MNSD. While STDP translates the axis of the cylinder (ζ, shown in dashed gray), *L*_*d*_ varies its radius. For this figure we assumed that the multi-neuronal patterns arrive to the MNSD when the neurons are at their resting potentials.

In some scenarios, we aim at a certain tolerance to a temporal jitter of the input spikes. By changing the decay constant *L*_*d*_ we can modulate the tolerance of the structure: the higher (lower) the *L*_*d*_, the more selective (robust) the structure becomes to the jitter. Another relevant characteristic is that, when using the MNSD, the detection does not depend on the arrival time of the first spike but only on the intervals between spikes. In a three dimensional feature problem (characterized by three neuronal pathways), the corresponding hypervolume is (in our case, where all *L*_*d*_ are equal) a cylinder whose radius depends on *L*_*d*_ and its axis ζ has a slope of 45° with respect of each of the axes (see Figure 7). Its mathematical form is defined by the following expression:

(22) $$\begin{array}{l} \zeta =(t_{off\mathrm{set}}+\frac{1}{w(D_{1},ES_{1})-1},t_{offset}+\frac{1}{w(D_{2},ES_{2})-1},t_{offset}\\ \;+\frac{1}{w(D_{3},ES_{3})-1}) \end{array}$$

Where *t*_*offset*_ is the time of arrival of the first pulse of the sequence. In Figure 7 we represent the cylinder defined by our MNSD. If the arrival times of a pattern fall into the cylinder, the MNSD produces a spike.

## 3. Results

In order to show how the developed MNSD tool can be used to tackle pattern recognition problems, we test the structure on two different datasets, *dataset* 1 and *dataset 2*. Dataset 1 has been artificially generated, to show the behavior of the tool. Dataset 2 consists of real brain data, related to the recognition of cognitive states. In order to set up the MNSD, we implement Equations 2.3.2 and 2.3.3 in Matlab® environment. Taking into account the constrains for the correct operation of the MNSD, in the following simulations we initialize the structure as follows:

- *t*_*f, max*_ larger than the maximum possible Δ*t*_*in*_. To achieve this, we set *c* = 0.04 (i.e., *t*_*f, max*_ = 25 ms) and adapt the patterns employed in the simulations to fall within the range [0–25] ms;
- Input amplitudes are chosen to leed *D*_*n*_ around the center of the latency range (i.e., *t*_*f, max*_/2 = 12.5 ms), to obtain the largest margin to set *D*_*n*_. To achieve it, we set *A*(*ES*_*n*_) = 1 and *w*(*D*_*n*_, *ES*_*n*_) = 1.08; for simplicity, the other weights of the structure have also been set to the value 1.08;
- Weak lateral contributions;
- For the STDP we set τ_+_ and τ_−_ to a value that allows the interplay of spikes of the same sequence, but at the same time avoids interaction between adjacent sequences (τ+ = τ_−_ = 9.6 for both the datasets), and take *A*_+_ and *A*_−_ in a range that prevents abrupt changes of weights during the presentation of patterns, and at the same time show a progressive stabilization of the synaptic weights during the learning phase. We fix *A*_+_ = −*A*_−_ for simplicity, with their absolute value < 0.01 for both the datasets (specific values are given in the next subsections).

### 3.1. Dataset 1

**Figure 8:**
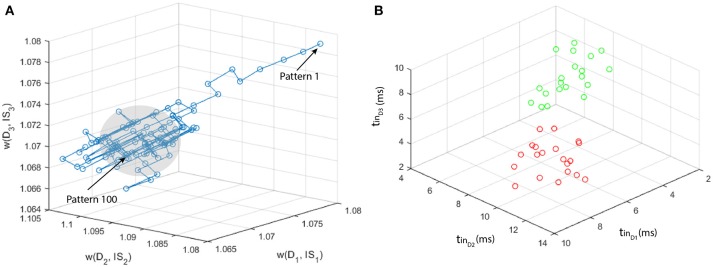
Example of simulation process, considering *L*_*d*_ = 0.37 and *D*_*c*_ = 5: **(A)** path of the weights along the presentation of the 100 patterns pertaining to class 1. A progressive stabilization of the weights is clearly noticeable (gray area) along the learning phase; **(B)** plot of the multi-neuronal spike patterns used for the test (instances of class 1 are depicted in green, instances of class 2 in red).

**Figure 9:**
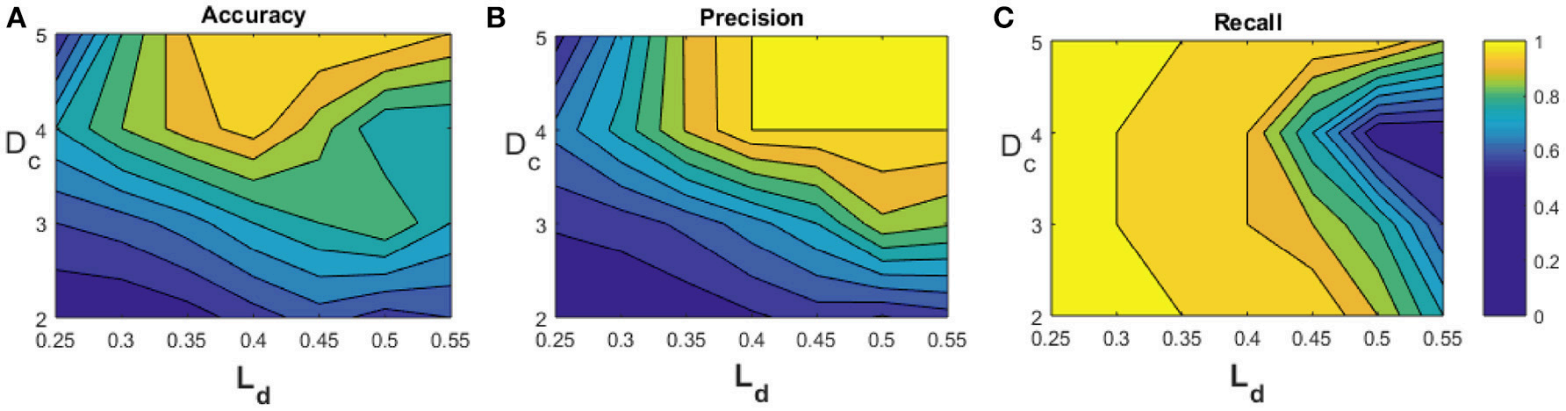
Test outcomes: **(A)** accuracy, **(B)** precision, and **(C)** recall.

We generate 2 sets of multi-neuronal spike patterns (120 patterns for class 1 and 20 for class 2). We assume that the patterns are characterized by three features, so that we generate 3 vectors per class, using Matlab ®. Vector values are generated with unitary standard deviation σ, and mean μ such that the centroid of the point set of class 2 is located at a certain distance *D*_*c*_ to the axis ζ identified by the point set of class 1 (otherwise the two point sets would represent the same pattern, as explained in section 2.3.3). Using a classifier based on a single MNSD, we carried out a set of simulations varying over a broad range of values the parameters *D*_*c*_ and *L*_*d*_ ([2-5] and [0.25-0.55], respectively). With regards to the STDP we choose *A*_+_ = −*A*_−_ = 0.002. The MNSD was trained with 100 samples of class 1, in order to recognize the distinctive timings of this class. For the test phase we used 20 samples from class 1 and 20 samples from class 2. During the training phase the structure adjusted its weights due to plasticity effects while during the test phase the weights were kept constant. The target neuron should produce a spike only when the class 1 is detected, allowing us to differentiate between the two classes. For each set of simulations we have noticed that while unseen patterns were presented to the MNSD the weights moved through a trajectory depicted in Figure 8, achieving a progressive stabilization toward a combination of values that maximized the synchrony to the targets corresponding to the class 1 patterns. In Figure 8 we summarize the simulations process, whereas in Figure 9 we present the obtained results, where we consider the following indices:

(23) $$Accuracy=\frac{TP+TN}{TP+TN+FP+FN}$$

(24) $$Precision=\frac{TP}{TP+FP}$$

(25) $$Recall=\frac{TP}{TP+FN}$$

where TP stands for true positive, TN for true negative, FP for false positive and FN for false negative. As expected, the figure shows that the larger the *D*_*c*_, the better the classification performance. Differently *L*_*d*_ should be large enough to include in the hypervolume most of the points of the class to be recognized, but small enough not to include the points of class 2; in this example we found *L*_*d*_ = 0.37 as optimal value.

### 3.2. Dataset 2

The second dataset is related to the recognition of cognitive states, using real data from a motor-inhibitory (Go/NoGo) task (Falkenstein et al., 1999; López-Caneda et al., 2017). Such paradigm is useful to study neural substrates of response inhibition and sustained attention processes. *Event-related potentials* studies have found discriminative neuroelectric components (e.g., *N*2 and *P*3, Eimer, 1993; Falkenstein et al., 1999; Falkenstein, 2006) between target and non-target conditions, evidencing inhibition functional networks and different motor responses (Kamarajan et al., 2004; Lavric et al., 2004; Pandey et al., 2012).

The two classes of stimuli were presented to 67 participants (age range: 13–15 years old), consisting in blue squares/green circles as targets (Go) and green squares/blue circles as non-targets (NoGo), displayed randomly and with a 70/30 presentation ratio. Participants were instructed to press a button as fast as possible only when a target was shown in the center of the screen (with the right hand Go and the left hand for NoGo). The stimuli were presented for 100 ms with a *stimulus onset asynchrony* (time interval between two trials) of 1400±200 ms.

High-density MEG signals were obtained from 306 channels (102 pairs of planar gradiometers and 102 magnetometers) with an Elekta Neuromag Vectorview system situated in a magnetically and electrically shielded room. Only the 102 Magnetometers were used to carry out the analysis. The signals were recorded with a 1000 Hz sampling rate and filtered online with a band pass 0.1–330 Hz filter. A *3Space Isotrak II* system was used for the registration of the magnetic coil positions, fiduciary points, and several random points spread across the participant scalp. For this preliminary study, we have chosen randomly one of the participants that performed this task. Methods were carried out in accordance with the approved guidelines and general research practice. The study was approved by the ethical committee of the Complutense University of Madrid. Informed consent has been obtained from the parents (or guardians) of the subjects, since they are under the age of 16.

**Figure 10:**
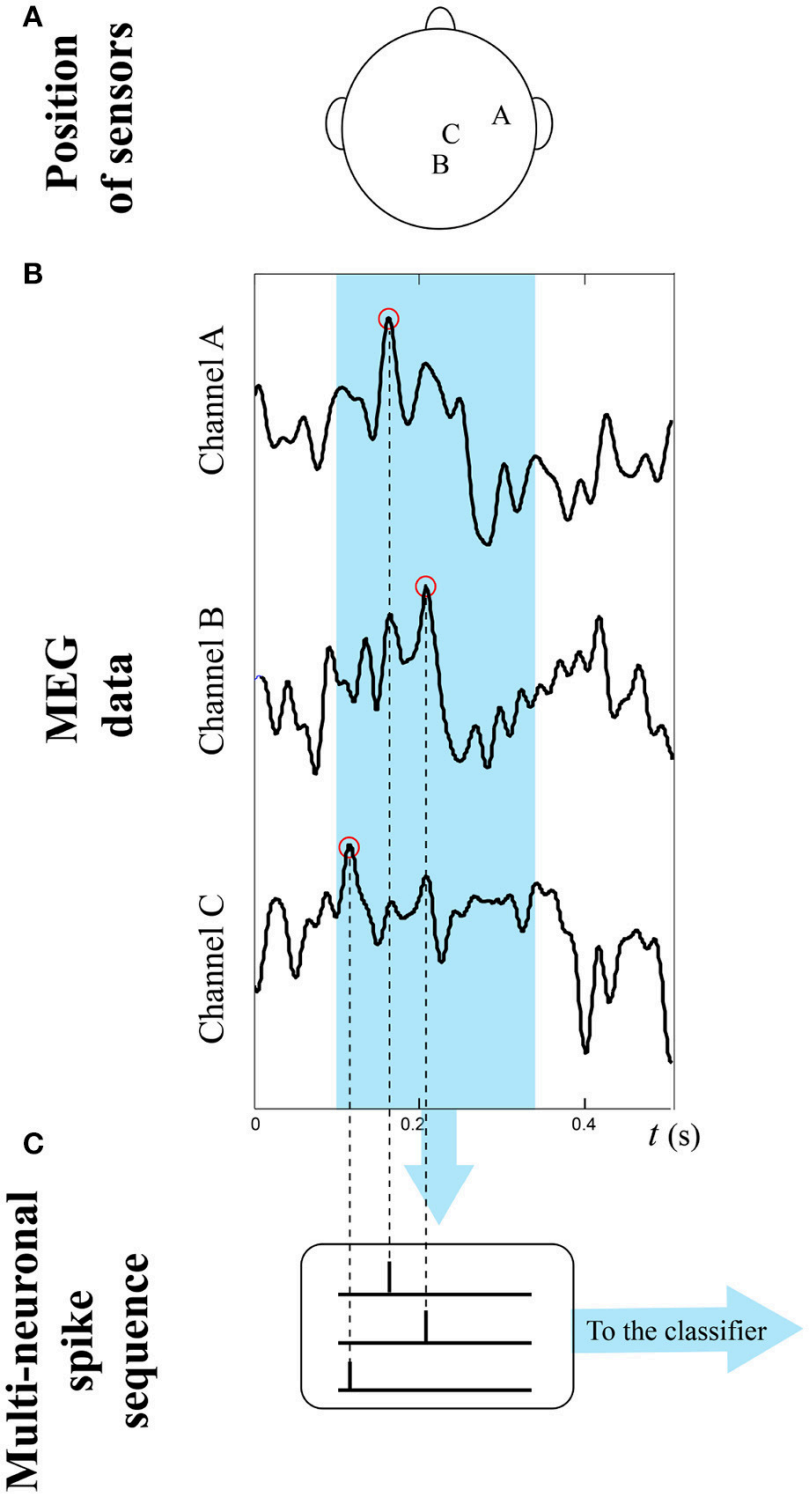
Multi-neuronal spike sequence generation process.**(A)** Position of channels A,B,C; **(B)** extraction of the time series from a single trial: only signals deriving from three channels are considered; **(C)** maxima are selected to generate multi-neuronal spike sequences.

MEG data was first visually inspected to exclude obvious artifacts. Although a statistical test revealed clear differences between the two conditions on a sufficiently large set of samples, neural noise renders the trial-specific discrimination between the two classes of responses not trivial. To overcome this limitation, we extracted in each trial the segment in the time interval [0.1, 0.35]*s* after the stimulus presentation to exclude the premotor response (which starts around 400 ms) (Deecke et al., 1976; Ikeda et al., 2000). This ensures that the activity is related to the cognitive task only, reducing the artifacts due to the motor action. We performed a second statistical test to select the three channels that best differentiate the responses of the two classes: (1) from the time series of each trial and sensor we extracted the maximum peaks along the mentioned time interval and transformed them into spike sequences; (2) for each possible triad of sensors we generated the related point set in the feature space, and selected the triad of sensors which point set presents, at the same time, the higher *D*_*c*_ and the lowest variability along the trials. In this way we selected the representative sensors 1,331, 2,021, and 2,231 (that we call channel A, channel B, and channel C, respectively) as the most significant ones (see Figure 10). We realized a classifier based on a single MNSD trained to recognize the distinctive timings of the NoGo class, considering 105 NoGo samples of the used dataset for the learning. For the test phase we used both Go and NoGo samples (22 Go and 22 NoGo). During the training phase the structure adjusted its weights due to plasticity effects while during the test phase the weights were kept constant. Once trained, the target neuron should produce a spike only when the NoGo class was detected, allowing us to differentiate between Go and NoGo classes. To make the structure compatible with this problem, we scaled the 250 ms interval of the segments by a factor 10 (obtaining sequences of 25 time units). Starting from the setting obtained from the previous example, we varied the following 2 parameters to optimize the structure: *A*_+_ and *A*_−_ to obtain the stabilization of synaptic weights after the training phase, and *L*_*d*_ to confer the proper tolerance to the structure. We found *A*_+_ = −*A*_−_ = 0.0085 and *L*_*d*_ = 0.13 as optimal values.

While new patterns were presented to the MNSD, the weights moved through a trajectory toward a combination of values that maximized the synchrony to the targets corresponding to the NoGo patterns. In Table 1 we report the details of the test performed on the trained MNSD, which classification performances are summarized below:

- Accuracy = 0.68
- Precision = 0.68
- Recall = 0.68

**Table 1:** Test results.

		**Predicted values**	
		**Positive**	**Negative**
**Real values**	**Positive**	15	7
	**Negative**	7	15

*Since the MNSD is trained to recognize the NoGo patterns, positives represent the NoGo trials, whereas negatives the Go trials*.

**Figure 11:**
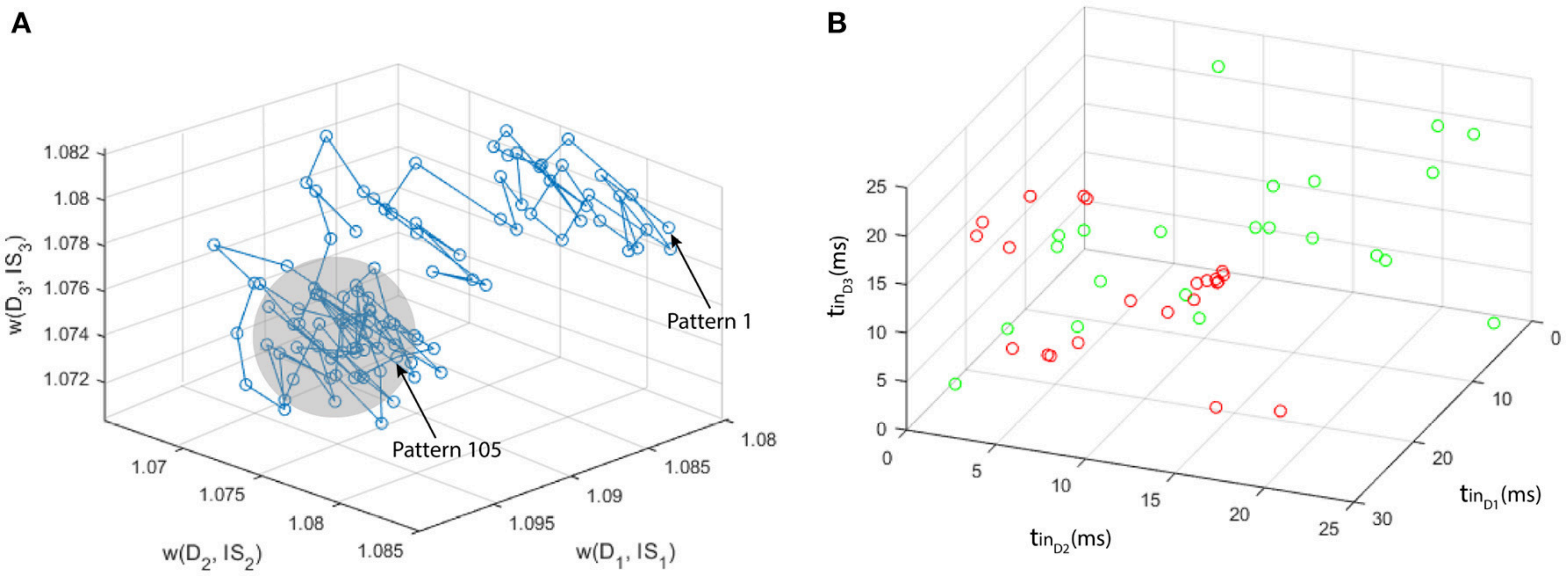
Resume of the simulation process: **(A)** path of the weights along the presentation of the 105 patterns pertaining to class 1. A progressive stabilization of the weights is clearly noticeable (gray area) along the learning phase. **(B)** plot of the multi-neuronal spike patterns used for the test (instances of Go are depicted in green, instances of NoGo in red).

In Figure 11 we schematize the simulation process, showing the path of the weights along the presentation of the 105 multi-neuronal spike patterns of the NoGo class, and the plot of the 44 patterns used for the test. It can be seen that the NoGo class is not easily separable using only 3 dimensions (MEG channels), justifing the modest classification performance.

**Figure 12:**
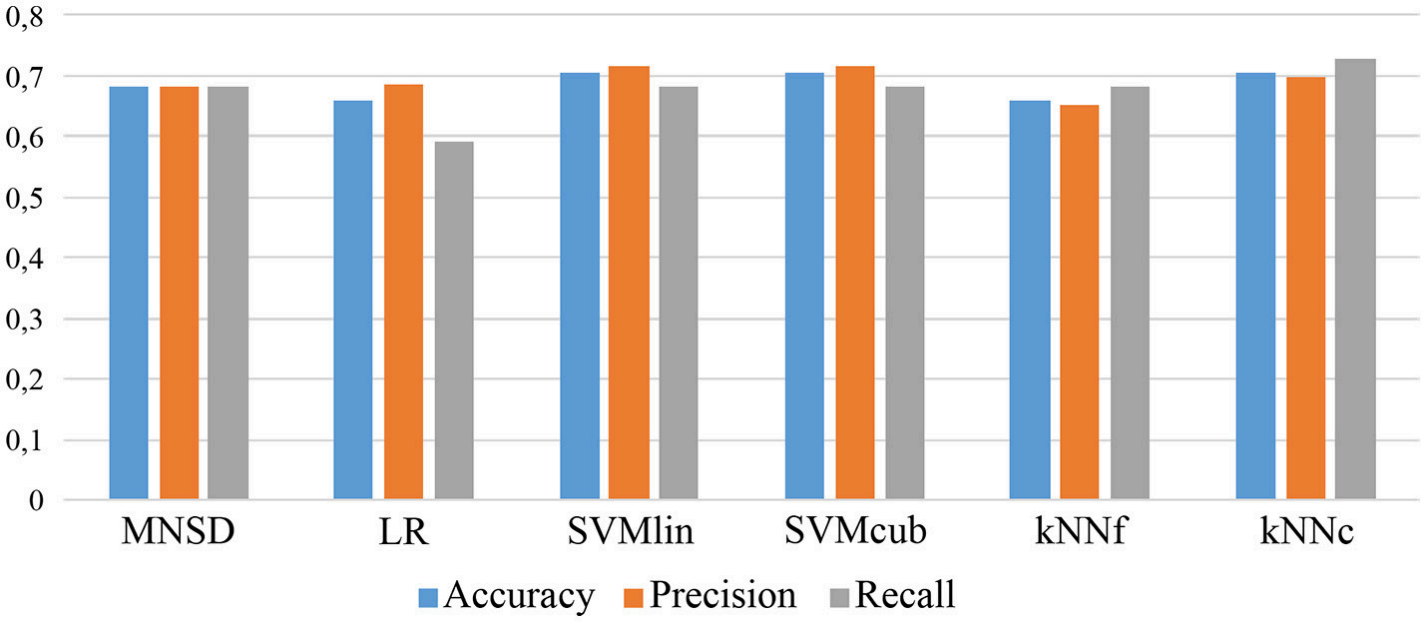
Comparison with other classification techniques: LR, SVM (with linear and cubic kernels) and kNN (fine and coarse, i.e., with 1- and 100-neighbors respectively).

Finally, in order to show the validity of our method, we have compared the MNSD with other classification methods: *logistic regression* (LR), *support vector machine* (SVM) and *k-Nearest neighbors* (kNN). The latter classifiers have been implemented and trained using the *Matlab classification learner* ® toolbox. The MNSD shows similar performances with respect to the other classification techniques, reporting better results than logistic regression and kNN (one-neighbor type). The comparison is summarized in Figure 12 in terms of classification performances.

## 4. Discussion and conclusions

In this study we have presented a multi-neuronal spike-pattern detection structure, MNSD, which combines the LIFL neuron model and heterosynaptic STDP, to perform online learning and recognition of multi-neuronal spike patterns.

The presented structure includes a bio-plausible self-tuning mechanism, which is able to learn and recognize multi-neuronal spike sequences through repeated stimulation. The time-amplitude conversion operated by the spike latency feature is one of the key operation principles of the structure, then the same task could not be performed by a simple LIF. Heterosynaptic excitatory STDP is allowed by the lateral connections in the network. It represents a mechanism to enhance synaptic transmission, or synapsis strengthening, and consequently the sensitivity to incoming sensory inputs (Christie and Westbrook, 2006).

To illustrate the ability of our structure, we have used the MNSD tool to discriminate between Go and NoGo decision during a motor-inhibitory task, and compared it to other classification methods, obtaining good results. MNSD can be further applied to problems with a greater number of features, and to other contexts of temporal stream data where SNN have already been applied (Lo Sciuto et al., 2016; Brusca et al., 2017).

STDP is present in different areas of the brain, including sensory cortices such as the visual and auditory, as well as the hippocampus (Matsumoto et al., 2013; Yu et al., 2013, 2014). Since STDP associates with coincidence detectors, where neurons get selective to a repetitive input pattern, it is thought to be crucial for memory and learning of the attributes of the stimuli (e.g., visual and auditory stimuli), even when the exposure is to meaningless sensory sequences that the subject is unaware of (Masquelier, 2017). Thus, the structure presented here may help understanding how humans learn repeating sequences in sensory systems. In fact, in sensory systems, different stimuli evoke different spike patterns but the exact way this information is extracted by neurons is yet to be clarified.

We can envisage to expand our MNSD structure in a modular way, such that each class is topologically structured with elementary building blocks among repetitive cortical columns and microcircuits: add other branches in parallel to increase the number of features, or inject the same *ES* to more than one delay neuron to obtain articulated shapes of *class hypervolumes*.

In the literature there are many learning methods for SNNs that make use of biologically plausible strategies. While the most of methods are based on synaptic learning rules aimed at modifying the weights (i.e., *weight adjustment*) only few of them consider also the modulation of the delay time to achieve learning (i.e., *delay shift* Brückmann et al., 2004; Adibi et al., 2005; Taherkhani et al., 2015; Matsubara, 2017; Hwu et al., 2018). It has been demonstrated that the alteration of delays has advantages in forming spatiotemporal memories, over altering synaptic weights (Izhikevich, 2006; Hwu et al., 2018). Various biological justifications have been attributed to the delay adjustment process, among which the activity-dependent myelination, which in turn results in the modulation of conduction velocities (Fields, 2008, 2015; Matsubara, 2017; Hwu et al., 2018). Differently, MNSD integrates the delay and weight adjustment methods by means of the well-known mechanism of spike latency (Izhikevich, 2004), representing a new opportunity to understand the mechanisms underlying biological learning.

## Data availability statement

The dataset generated for this study can be found in the GitHub repository of the *Laboratory of Cognitive and Computational Neuroscience* (LCCN): [https://github.com/LCCN].

## Author contributions

GS designed the model and the computational framework. GS, EP, and CM designed the experiment. GS, LA, LC, ML, FM, CM, and EP wrote the paper. FM and LA provided and analyzed brain data. ML contributed to shape the experiment.

### Conflict of interest statement

The authors declare that the research was conducted in the absence of any commercial or financial relationships that could be construed as a potential conflict of interest.
