# Supplementary material for: A Neuro-Inspired System for Online Learning and Recognition of Parallel Spike Trains, Based on Spike Latency, and Heterosynaptic STDP
Source: Front Neurosci. 2018 Oct 31;12:780. doi: 10.3389/fnins.2018.00780 (PMC6220070; doi:10.3389/fnins.2018.00780)
Supplement: Supplementary file 1 [file Data_Sheet_1.PDF]

Supplementary Material: Online learning and recognition of multineuronal spike patterns, based on spike latency and heterosynaptic STDP

1 T_r CALCULATION

Referring to Fig. S1, at the time the neurons inner state is altered from a second input (here excitatory, but non influencial to calculation purposes), the *intermediate state* S_i is determined, and then T_r is calculated.

Using the event-driven simulation technique the update of network elements happens only when they receive or emit a spike. Once an input spike arrives in active mode, the S_i is calculated on the basis of the time remaining to the spike generation.

Referring to the generic inner state S_i the firing equation is:

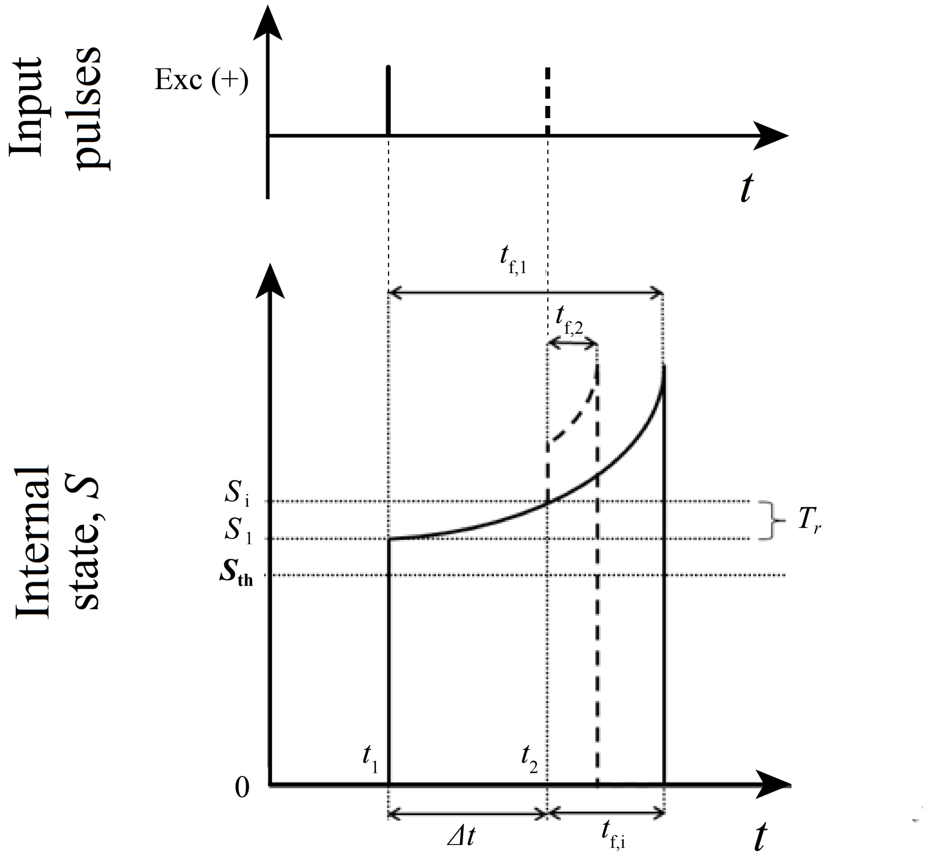

Figure S1. Representation of T_r. LIFL neuron in active mode is characterized by a spontaneous growth of S. If a pulse arrives before the actual spike generation, S is modified and the t_f will be recalculated. The recalculation considers the intermediate state S_i, i.e., the neuron state at the time the pulse arrives.

$$t_{f,i} = \frac{1}{S_i - 1} \tag{S1}$$

We define:

$$\Delta t = t_2 - t_1 \tag{S2}$$

where t_1 and t_2 represent the arrival instants of the synaptic pulses to the considered neuron. Then:

$$t_{f,i} = t_{f,1} - \Delta t \tag{S3}$$

Rearranging Eq. S1, we obtain:

$$S_i = \frac{1}{t_{f,i}} + 1 \tag{S4}$$

Now we combine Eq. S3 with Eq. S4

$$S_i = \frac{1}{t_{f,1} - \Delta t} + 1 \tag{S5}$$

By defining

$$T_r = S_i - S_1 \tag{S6}$$

where

$$S_1 = \frac{1}{t_{f,1}} + 1 \tag{S7}$$

and putting Eq .S5 and S7 in S6, we obtain:

$$T_r = \frac{1}{t_{f,1} - \Delta t} - \frac{1}{t_{f,1}} \tag{S8}$$

that can be rearranged as

$$T_r = \frac{\Delta t}{t_{f,1}(t_{f,1} - \Delta t)} \tag{S9}$$

Note that we are interested in determining an intermediate state; this implies that we consider the second synaptic pulse only if its timing (i.e., t_2) falls before the spike occurs. This gives us:

$$\Delta t < t_{f,1} \tag{S10}$$

thus we do not have restrictions from the denominator of S9.

The relation S9 can be generalized to the case as more input modify the firing time; then, we can write

$$T_r = S_{ic} - S_{ip} = \frac{\Delta t_i}{t_{f,ip}(t_{f,ip} - \Delta t_i)} \tag{S11}$$

with

$$\Delta t_i = t_{ic} - t_{ip} \tag{S12}$$

where the subscript ip stays for *intermediate-previous* and ic for *intermediate-current*.

We can also make explicit the dependence of T_r from the previous state, by inverting $t_{f,ip}$ trough Eq. S1, obtaining:

$$T_r = \frac{(S_{ip} - 1)^2 \Delta t}{1 - (S_{ip} - 1)\Delta t} \tag{S13}$$

Obviously, the same considerations on the arrival time of the second pulse remain valid, thus we do not have restrictions imposed by the denominator of S13.

2 CONDITIONS FOR THE SPIKE GENERATION AT THE TARGET NEURON

We hypothesize that contributions from delay neurons arrive "sufficiently synchronous" at the target (i.e., sufficiently synchronous to not allowing the internal state collapse to zero between two arrivals). Considering Fig.5 in the main text, the maximum value that is reached by the internal state of T is S_M, that is given by:

$$S_M = 3 - L_d \cdot \Delta t_{out,tot} \tag{S14}$$

where $\Delta t_{out,tot}$ is the time window between the first and the last spike reaching the target, evoked by a single sequence, equals to:

$$\begin{cases} \Delta t_{out,tot} = max(|\Delta t_{out_{D_1,D_2}}|, |\Delta t_{out_{D_1,D_3}}|) \ , & if \quad \Delta t_{out_{D_1,D_2}} \cdot \Delta t_{out_{D_1,D_3}} > 0 \\ \Delta t_{out,tot} = |\Delta t_{out_{D_1,D_2}}| + |\Delta t_{out_{D_1,D_3}}| \ , & if \quad \Delta t_{out_{D_1,D_2}} \cdot \Delta t_{out_{D_1,D_3}} < 0 \end{cases} \tag{S15}$$

Now, considering the system of equations that relates the arrival times of the three contributions to T (see Eq.13 in the main text), we can explicitly write Eq.S15 with respect to the input arrival times and weights afferent to D_n:

$$\begin{cases} \Delta t_{out,tot} = max(|\Delta t_{in_{D_1,D_2}} - \frac{1}{w(D_1,ES_1)-1} + \frac{1}{w(D_2,ES_2)-1}|, |\Delta t_{in_{D_1,D_3}} - \frac{1}{w(D_1,ES_1)-1} + \frac{1}{w(D_3,ES_3)-1}|) \ , \\ \quad if \quad (\Delta t_{in_{D_1,D_2}} - \frac{1}{w(D_1,ES_1)-1} + \frac{1}{w(D_2,ES_2)-1}) \cdot (\Delta t_{in_{D_1,D_3}} - \frac{1}{w(D_1,ES_1)-1} + \frac{1}{w(D_3,ES_3)-1}) > 0 \\ \Delta t_{out,tot} = |(\Delta t_{in_{D_1,D_2}} - \frac{1}{w(D_1,ES_1)-1} + \frac{1}{w(D_2,ES_2)-1}) - (\Delta t_{in_{D_1,D_3}} - \frac{1}{w(D_1,ES_1)-1} + \frac{1}{w(D_3,ES_3)-1})| \quad , \\ \quad if \quad (\Delta t_{in_{D_1,D_2}} - \frac{1}{w(D_1,ES_1)-1} + \frac{1}{w(D_2,ES_2)-1}) \cdot (\Delta t_{in_{D_1,D_3}} - \frac{1}{w(D_1,ES_1)-1} + \frac{1}{w(D_3,ES_3)-1}) < 0 \end{cases} \tag{S16}$$

Then, given the specific input sequence, S_M should result greater than $1 + d$ in order to ensure that T generates a spike. Considering Eq.S14, we can then write the condition of activation of the target:

$$\Delta_{out,tot} < \frac{2-d}{L_d} \tag{S17}$$

In order to make Eq. S17 explicit with respect to the input weights and spike intervals, we can state that if $\Delta t_{in_{D_1,D_2}}$ and $\Delta t_{in_{D_1,D_3}}$ have a concordant sign, the following relation ensures that T spikes:

$$max(|\Delta t_{in_{D_1,D_2}} - \frac{1}{w(D_1,ES_1)-1} + \frac{1}{w(D_2,ES_2)-1}|, |\Delta t_{in_{D_1,D_3}} - \frac{1}{w(D_1,ES_1)-1} + \frac{1}{w(D_3,ES_3)-1}|) < \frac{2-d}{L_d} \tag{S18}$$

On the contrary, if $\Delta t_{in_{D_1,D_2}}$ and $\Delta t_{in_{D_1,D_3}}$ have discordant sign, then:

$$|(\Delta t_{in_{D_1,D_2}} - \frac{1}{w(D_1,ES_1)-1} + \frac{1}{w(D_2,ES_2)-1}) - (\Delta t_{in_{D_1,D_3}} - \frac{1}{w(D_1,ES_1)-1} + \frac{1}{w(D_3,ES_3)-1})| < \frac{2-d}{L_d} \tag{S19}$$
